# Self-Assembly of Supramolecular Architectures Driven by σ-Hole Interactions: A Halogen-Bonded 2D Network Based on a Diiminedibromido Gold(III) Complex and Tribromide Building Blocks

**DOI:** 10.3390/molecules27196289

**Published:** 2022-09-23

**Authors:** M. Carla Aragoni, M. Francesca Cherchi, Vito Lippolis, Anna Pintus, Enrico Podda, Alexandra M. Z. Slawin, J. Derek Woollins, Massimiliano Arca

**Affiliations:** 1 Dipartimento di Scienze Chimiche e Geologiche, Università degli Studi di Cagliari, S.S. 554 bivio per Sestu, 09042 Monserrato, Italy; 2 Centro Servizi di Ateneo per la Ricerca (CeSAR), Università degli Studi di Cagliari, S.S. 554 bivio per Sestu, 09042 Monserrato, Italy; 3 EaStCHEM School of Chemistry, University of St. Andrews, North Haugh, St. Andrews, Fife KY16 9ST, UK; 4 Department of Chemistry, Khalifa University, Abu Dhabi 127788, United Arab Emirates

**Keywords:** gold, supramolecular, halogen bond, sigma-hole, crystallography, DFT, polyhalide, bromine

## Abstract

The reaction of the complex [Au(phen)Br_2_](PF_6_) (phen = 1,10-phenanthroline) with molecular dibromine afforded {[Au(phen)Br_2_](Br_3_)}_∞_ (**1**). Single crystal diffraction analysis showed that the [Au(phen)Br_2_]^+^ complex cations were bridged by asymmetric tribromide anions to form infinite zig-zag chains featuring the motif ···Au–Br···Br–Br–Br···Au–Br···Br–Br–Br···. The complex cation played an unprecedented halogen bonding (XB) donor role engaging type-I and type-II XB noncovalent interactions of comparable strength with symmetry related [Br_3_]^−^ anions. A network of hydrogen bonds connects parallel chains in an infinite 2D network, contributing to the layered supramolecular architecture. DFT calculations allowed clarification of the nature of the XB interactions, showing the interplay between orbital mixing, analyzed at the NBO level, and electrostatic contribution, explored based on the molecular potential energy (MEP) maps of the interacting synthons.

## 1. Introduction

The assembly of solid-state architectures by exploiting both covalent and noncovalent interactions between the desired synthons is a challenging goal of supramolecular chemistry [1,2]. The reagents involved can be prepared by using conventional synthetic methods and subsequently reacted, thus triggering a sequence of multiple recognition steps: an initial supramolecular event or chemical reaction generates small units that can subsequently act as building blocks in a second supramolecular event and assemble to form aggregates displaying higher order [3]. This approach is typically followed when polyhalides are involved in the rational design and subsequent construction of supramolecular networks. As far as polyiodides are involved, extended and discrete species, with the general formula [I_2*m*+*n*_]*^n^*^−^ (*n*, *m* > 0), such as [I_4_]^2^^−^, [I_5_]^−^, and [I_7_]^−^, can be identified [4]. The largest discrete polyiodide has been reported in ferrocenium salt of [I_29_]^3−^ [5], showing a three-dimensional (3D) network built of [I_5_]^−^, [I_12_]^2^^−^, and I_2_ units. All polyiodides can be considered as formed by secondary bonds involving I^−^, I_2_, and [I_3_]^−^ building blocks, with $d_{I\cdot \cdot \cdot I}$ distances shorter than 3.6–3.7 Å [6,7]; polyiodides further interact through solid-state contacts with distances up to the sum of van der Waals (vdW) radii (2 · $r_{I}^{vdW}$= 4.20 Å), leading to the formation of extended or even infinite networks.

The ability of halides to catenate decreases on passing from iodine to bromine and chlorine [6]. Therefore, the number of reported discrete and extended polybromides [8] is much lower than that of polyiodides [9]. An analysis of the structural features of [X1–X2–X3]^−^ three-body systems (X = Cl, Br, I) demonstrated that the *d*_X1-X2_ and *d*_X2-X3_ interatomic distances are correlated, and a relationship exists between the relative standardized elongations *δ*_X1–X2_ and *δ*_X2–X3_ [$\delta_{X1–X2}=(d_{X1–X2}-\left(r_{X1}^{cov}+r_{X2}^{cov}\right))/\left(r_{X1}^{cov}+r_{X2}^{cov}\right)$; $\delta_{X2–X3}=(d_{X2–X3}-\left(r_{X2}^{cov}+r_{X3}^{cov}\right))/\left(r_{X2}^{cov}+r_{X3}^{cov}\right)$; $r_{X}^{cov}$ = covalent radii of X], regardless of the nature of the halogen X [10]. Authentic polybromides feature Br–Br distances shorter than 3.2 Å, in accordance with what was empirically proposed by Maschmeyer [11], while Br···Br supramolecular contacts [12] can extend up to the sum of the relevant vdW radii (3.7 Å) [13]. Accordingly, a variety of polybromides have been reported to date, ranging continuously from [Br_3_]^−^ to [Br_11_]^−^ [11,14], as well as polybromide aggregates as large as [Br_20_]^2−^ (Br···Br distances 3.25–3.58 Å) [15] and [Br_24_]^2−^ (Br···Br distances 2.77–3.41 Å) [11]. A unique example of an infinite 2D polybromide network featuring all Br–Br distances shorter than 3.2 Å was reported by researchers in the compound {[Et_4_todit·2Br]^2+^(Br_2_)_2_(Br_2_)_3_}_∞,_ (Et_4_todit = 4,5,9,10-tetrathiocino-[1,2-*b*:5,6-*b'*]-1,3,6,8-tetraethyl-diimidazolyl-2,7-dithione) [16]. 

The nature of the interaction between the building blocks in polyhalides has been the subject of a vivid debate as a part of the wider discussion on Halogen Bonding (XB), defined as the attractive interaction between an XB donor R–X (R = heteroatom, metal ion, organic group; X = halogen) and an XB acceptor A in R–X···A systems [17,18]. Along with a “covalent” interpretation (A→X–R) based on orbital mixing resulting in a Charge-Transfer (CT) interaction [19], and showing an often-relevant π-contribution [20], the electrostatic σ-hole approach has been largely adopted [21]. According to this view, the interaction would be driven by the anisotropy of the electrostatic potential at the interacting atoms, the depletion of which, called σ-hole and representing an electrophilic region on the X atom, is typically disposed opposite to the covalent R–X bond of the donor group. Theoretical investigations indeed revealed that XB and other sister interactions, such as a hydrogen bond (HB) and chalcogen bond (ChB) [22], can be decomposed into an electrostatic and an orbital-mixing term [23], and that dispersion [24] also often plays a fundamental role [25,26]. 

Countercations play a fundamental role in templating the architectures of supramolecular polyhalide networks [16,27,28], and cationic metal complexes have been occasionally reported as counterions of extended polyhalide architectures [29,30], with some examples of halido gold complexes forming supramolecular networks based on halogen···halogen interactions [31,32]. Although the double salts of diimine-dichlorido gold(III) complexes show interactions based on aurophilic Au···Au [33,34] and Au···Cl interactions [35], due to the scarce tendency of Cl to originate Cl···Cl interactions, these complexes are unsuitable synthons for extended XB interactions. On the contrary, the less common bromido gold(III) complexes are, in principle, promising building blocks for supramolecular networks, due to their stability accompanied by the tendency of bromine to form soft···soft interactions [11,14,27]. As a proof of concept, we report here on the first example of a tribromide salt of a diimine dibromido gold(III) complex originating an infinite 2D network based exclusively on σ-hole interactions.

## 2. Results and Discussion

**Figure 1:**
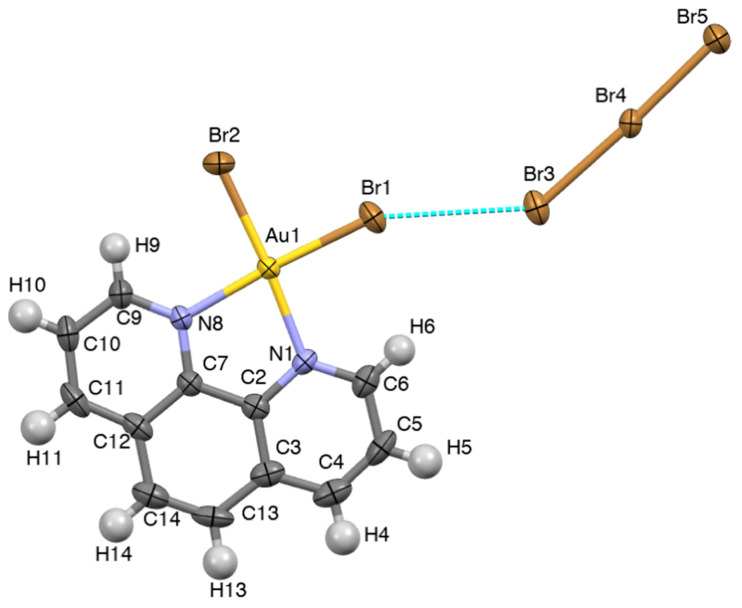
Molecular view of compound **1** laying on the *ac* plane. Ellipsoids were drawn at 60% probability level.

The reaction of [Au(phen)Br_2_](PF_6_) (phen = 1,10-phenanthroline; Scheme S1) with molecular dibromine in MeCN led to small orange platelet-shaped single crystals shown by X-ray diffraction analysis to be {[Au(phen)Br_2_](Br_3_)}_∞_ (**1**). The stoichiometry of compound **1** recalled that of the complex [Au(dppmS)AuBr_2_](Br_3_), obtained by dibromine oxidation of the gold(I) neutral complex Au(dppmS)Br [dppmS = bis(diphenylphosphino)methane sulfide] [36]. In the case of **1**, the formation of the tribromide ion, clearly testifying to a partial reduction of dibromine, could be possibly attributed to solvolysis or reaction with incipient moisture, as previously reported in the case of reactions between related polypyridine derivatives and molecular dihalogens [37]. In the complex cation [Au(phen)Br_2_]^+^ (Figure 1), the metal ion showed a *pseudo*-square-planar coordination geometry, with Au–Br bond lengths of 2.3825(7) and 2.3853(8) Å, very close to those found in the only other reported phenanthroline dibromido Au^III^ complex, namely [Au(Me_2_phen)Br_2_][AuBr_2_] [Me_2_phen = 5,6-dimethyl-1,10-phenanthroline; Cambridge Structural Database (CSD) code VEQZUP; Au–Br = 2.378(4) and 2.404(5) Å] [38]. The Au–N distances [2.074(4) and 2.077(4) Å] were close to those featured by [Au(Me_2_phen)Br_2_][AuBr_2_] [2.05(4) and 2.01(3) Å] and the average value [2.057(12) Å] calculated for the 15 cases deposited at the CSD of [Au(**L**)Cl_2_]^+^ complex cations featuring a 1,10-phenanthroline derivative **L** [39]. 

**Figure 2:**
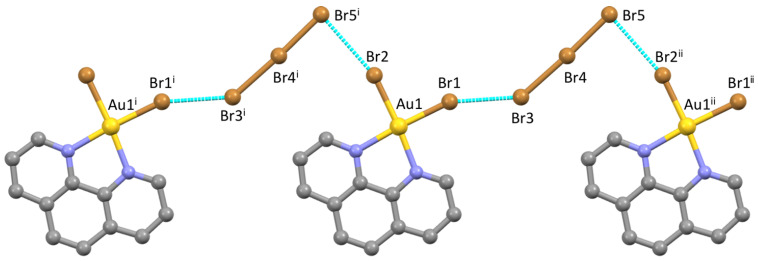
Au–Br···Br XB interactions resulting in a zig-zag infinite chain running along the *c* axis. ^i^ = x, y, −1 + z; ^ii^ = x, y, 1 + z. Hydrogen atoms were omitted for clarity.

The tribromide anion showed structural features [Br3–Br4, 2.6022(9); Br4–Br5, 2.4871(9) Å; Br3–Br4–Br5, 177.32(3) °] close to the average values calculated for isolated tribromide anions deposited at the CSD [40] and falling within the structural correlation typical for Br–Br–Br three-body systems ($\delta_{\mathrm{Br}3–\mathrm{Br}4}$ = 0.141; $\delta_{\mathrm{Br}4–\mathrm{Br}5}$ = 0.091; Figure S1) [10]. The terminal bromido anions bound to the Au^III^ metal ion in the complex cation were engaged in weak XB interactions with two symmetry-related asymmetric linear tribromide anions (Br1···Br3, 3.378; Br2···Br5^i^ = 3.403 Å; ^i^ = x, y, −1+z; Figure 2) laying on the same molecular plane. The Br1···Br3 interaction was remarkably bent (Br1···Br3–Br4, 141.54°), while the Br2···Br5^i^ one was almost orthogonal (Br2···Br5^i^–Br4^i^, 86.71°). The Br···Br interactions defined a zig-zig Au–Br···Br–Br–Br···Au–Br··· infinite motif developed along the *c* axis of the monoclinic crystal system (Figure 2), featuring Br···Br distances close to those displayed by the solid-state Br_2_ (3.31 and 3.79 Å) [41].

A network of weak [42] C–H···Br HB contacts (*d*_C···Br_ distances < 4.0 Å; *d*_H···Br_ < 3.0 Å; sum of H and Br vdW radii 3.05 Å) [13] joined the halogen-bonded chains to each other to form an infinite 2D network laying on the *ac* plane (Figure S2). Finally, weak contacts between the 1,10-phenanthroline ring and the central Br4 atom of tribromide anions of adjacent 2D layers contributed to building up a 3D supramolecular network made up of parallel layers spaced by *b*/2 (Figure S3).

**Figure 3:**
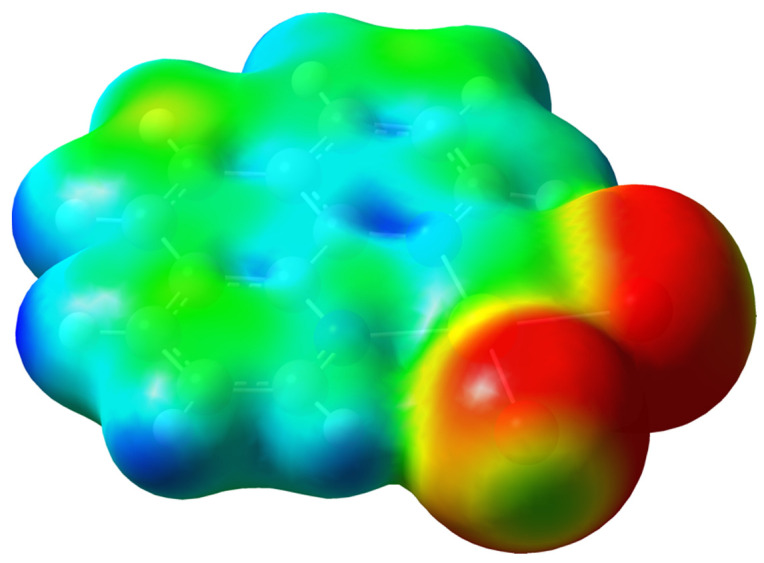
Potential electrostatic energy map [range 0.09 (red)–0.20 (blue) a.u.] calculated for the [Au(phen)Br_2_]^+^ cation at the DFT level on the density map (electron density = 9.0 · 10^−3^ |e|/Bohr^3^).

**Figure 4:**
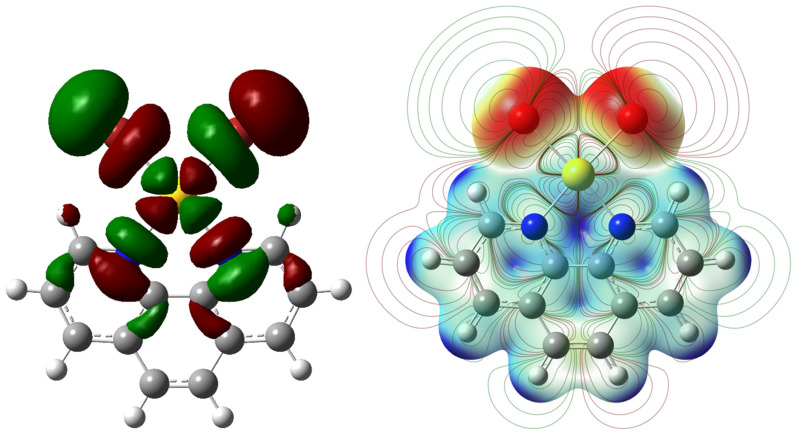
Isosurface of the BD* NBO #92 (left) and 2D isovalues projected over the potential electrostatic energy map [range 0.09 (red)–0.20 (blue) a.u.] calculated for the [Au(phen)Br_2_]^+^ cation at the DFT level on the density map (electron density = 9.0 · 10^−3^ |e|/Bohr^3^).

A Potential Energy Map calculated for the [Au(phen)Br_2_]^+^ cation, optimized at the DFT level, showed σ-holes located on the negatively charged bromido ligands (*Q*_Br1_ = *Q*_Br2_ = –0.118 |e|) opposite to the Au1–Br1 and Au1–Br2 bonds (Figure 2). These regions represented the electrophilic ends of the molecule, acting therefore as XB donor sites. A natural bond orbital (NBO) [43,44] analysis showed that the antibonding NBO (BD*) with respect to the Au–Br bonds was located along the same directions (Figure 3 and Figure 4). The [Br_3_]^−^ ion can act either as an electrophile when interacting via the terminal σ-holes (blue region in Figure S4) along the molecular axis, or as a nucleophile when interacting through its belt of negative electrostatic potential in a bent geometry (red region in Figure S4) [4].

Both the Au1–Br2···Br5–Br4–Br3 and the Au1–Br1···Br3–Br4–Br5 moieties could be considered as the result of XB noncovalent interactions. The Au–Br1···Br3–Br4 interaction, showing a flattened Z-shaped motif, could be considered as an unbalanced type-I XB (Figure S5, top) [17,45,46]. This motif could be envisaged in numerous cases, for example in interacting tribromides Br–Br–Br···Br–Br–Br forming Br–Br···Br angles in the range 144–160° and Br···Br distances in the range 3.239–3.494 Å [47,48,49,50]. The roughly orthogonal interaction between the Au1–Br2 bond and the Br5–Br4–Br3 tribromide, in agreement with the topology of the regions of positive and negative potentials on the two systems (Figure 2, Figure 4 and Figure S2), was instead a type-II XB (Figure S5, bottom) [17]. Both interactions were further analyzed at NBO level. A second order perturbation theory analysis (SOPTA) of the Fock matrix in the NBO basis revealed that the type-I interaction (5.25 kcal·mol^−1^ at the structural geometry) involved a charge transfer (CT) from two *p*-type lone pairs (LPs) of electrons on the Br3 atom (4*p* component: 88.8% and 95.9%) to the antibonding (BD*) natural orbital of the Au1–Br1 system (Figure 4) [51]. The type-II interaction (4.61 kcal·mol^−1^ at the structural geometry) was due to an overlap between one 4*p* LP (98.2%) on the Br5 atom of the tribromide anion and the antibonding orbital of the Au1–Br2 system (Figure 4). The [Au(phen)Br_2_](Br_3_) ensemble featuring the type-II interaction was successfully optimized as an isolated system. Although in this isolated ionic pair model, the interaction was forcedly overestimated (with a CT of 0.492 |e| from [Br_3_]^−^ to [Au(phen)Br_2_]^+^), the pattern of optimized metric parameters confirmed the previous analysis [optimized Au1–N1 distance, 2.087 and 2.252 Å for [Au(phen)Br_2_]^+^ and [Au(phen)Br_2_](Br_3_), respectively; Figure S6] [38,39]. The Br5→Br2–Au1 CT interaction (23.60 kcal·mol^−1^, Figure S6) weakened the Br5–Br4 bond within the [Br_3_]^−^ anion, determining a remarkable asymmetry in the tribromide. 

Notably, the LP→ σ^*^ CT found in type-I and type-II interactions in {[Au(phen)Br_2_](Br_3_)}_∞_ recalled those occurring in polyhalide anions featuring an L-shaped motif, such as [X_5_]^−^ ([X_3_]^−^···X_2_), [X_8_]^2−^ ([X_3_]^−^···X_2_··· [X_3_]^−^) and [X_7_]^−^(X_2_···[X_3_]^−^···X_2_), isolated for X = Br and I [6,7,11,14,52].

The comparison of the Br–Br structural bond lengths within the [Br_3_]^−^ anion (Br3–Br4 larger than Br4–Br5) and the Br···Br contacts (Br1···Br3 slightly shorter than Br2···Br5^i^) confirmed that the two types of interactions were of comparable strength, the type-I interaction being slightly stronger than the type-II one. The interplay of the structural effects of type-I and type-II XB Br···Br interactions determined the geometry of the polymeric chain in {[Au(phen)Br_2_](Br_3_)}_∞_ and hence the resulting 2D supramolecular network.

## 3. Materials and Methods

Commercial solvents (reagent-grade) and reagents were used without further purification. Melting points were determined on a FALC mod. C (up to 290 °C) apparatus. Elemental analyses were carried out with a CHNS/O PE 2400 series II CHNS/O elemental analyzer (T = 925 °C). FTIR spectra were recorded on a Thermo-Nicolet 5700 spectrometer at room temperature. KBr pellets with a KBr beam splitter and KBr windows (4000−400 cm^−1^, resolution 4 cm^−1^) were used. ^1^H-NMR measurements were carried out in CD_3_CN (stored under molecular sieves prior to use) at 25 °C, using a Bruker Advance 300 MHz (7.05 T) spectrometer operating at 300.13 MHz. Chemical shifts were reported in ppm (δ) and were calibrated to the solvent residue. X-ray single-crystal diffraction data for compound **1** (Tables S1–S3) were collected using a Rigaku XtaLAB P200 diffractometer equipped with a MoKα radiation and operating at 93 K. The data were indexed and processed using CrystalClear-SM Expert 2.1 b45. A multi-scan absorption correction was performed using REQAB. The structure was solved with the ShelXT Version 2018/2 [53] solution program using direct methods and using CrystalStructure 4.3 as the graphical interface. The model was refined with the ShelXL Version 2018/3 [54] using the full matrix least squares minimization on F^2^. The CCSD was accessed by means of Conquest 2022.1.0 (CSD version 5.43). The computational investigation on the complex cation [Au(phen)Br_2_]^+^, the tribromide anion [Br_3_]^−^, and the [Au(phen)Br_2_](Br_3_) system was carried out at the DFT level by adopting the Gaussian 16 [55] suite of programs. Following the results of previously reported calculations on related systems [56,57,58], the PBE0 [59] hybrid functional was adopted, along with the full-electron split valence basis sets (BSs) def2-TZVP [60] for light atomic species (C, H, N, Cl, and Br) and the CRENBL basis sets [61] with RECPs for heavier gold species. BS data were extracted from the EMSL BS Library [62]. Harmonic frequency calculations were carried out to verify the nature of the minima of the optimized geometry. Charge distributions were evaluated at the NBO level (Tables S5 and S8) [43,44,51]. The programs GaussView 6.0.16 [63] and Chemissian 4.53 [64] were used to investigate the optimized structures and molecular orbital shapes.

### Synthesis

KBr (0.3199 g, 2.688 · 10^−3^ mol) and an excess of KPF_6_ (3.1458 g, 1.7091 · 10^−2^ mol) were added to 25 mL of a KAuCl_4_ (0.2503 g, 6.624 · 10^−4^ mol) water solution. A MeCN solution of 1,10-phenanthroline (0.1754 g, 9.733 · 10^−4^ mol) was added dropwise. The mixture was magnetically stirred for 4 h, the resulting orange precipitate was filtered off, washed with small amounts of water, toluene, and diethyl ether, and dried under reduced pressure. The yield was 0.3587 g (73%). The M.P. was 239 °C (decomp.). The FTIR results were ṽ = 438 (vw), 557 (m), 660 (vw), 704 (m), 725 (vw), 740 (vw), 750 (w), 779 (w), 837 (s), 850 (s), 879 (w), 1003 (vw), 1103 (vw), 1113 (vw), 1153 (w), 1221 (vw), 1225 (vw), 1323 (vw), 1350 (w), 1412 (vw), 1435 (m), 1456 (w), 1522 (m), 1585 (w), 1604 (w), and 3091 (w) cm^−1^. The UV-Vis (MeCN) results were λ (ε) = 207 (4.54 · 10^4^), 223 (5.47 · 10^4^), and 281 nm (2.63 · 10^4^ M^−1^ cm^−1^). The ^1^H-NMR (300 MHz, CD_3_CN) were δ = 9.94 (s, 2H), 9.16 (dd, ^1^J_HH_ = 8.27 Hz, ^2^J_HH_ = 0.96 Hz, 2H), 8.38 (s, 2H), and 8.32 (dd, ^1^J_HH_ = 7.56 Hz, ^2^J_HH_ = 6.17 Hz, 2H). The elemental analysis [calcd. for [Au(phen)Br_2_](PF_6_)] data were C 20.99 (21.13), H 1.16 (1.18), and N 4.05 (4.11). An excess of molecular dibromine (2 drops) was added to 3 mL of a 9.94 · 10^−3^ M solution of [Au(phen)Br_2_](PF_6_) in MeCN. Small platelet-shaped crystals of {[Au(phen)Br_2_](Br_3_)}_∞_ (**1**) suitable for single-crystal X-ray diffraction were obtained by slow infusion of diethyl ether into the resulting solution.

## 4. Conclusions

In conclusion, the rearrangement, anion exchange, and subsequent solid-state self-assembly of the [Au(phen)Br_2_]^+^, Br_2_, and Br^−^ building blocks led to the formation of the infinite network {[Au(phen)Br_2_](Br_3_)}_∞_ (**1**), driven exclusively by noncovalent XB and HB interactions. The bromido ligands of the complex cation [Au(phen)Br_2_]^+^ acted as donors in type-I and type-II XB interactions with symmetry related [Br_3_]^−^ anions, thus leading to a rare example of a supramolecular architecture based on interacting halido gold(III) complex and polyhalide building blocks. The isolation of compound **1** clearly showed that bromido gold(III) complexes can behave as promising XB building blocks for the design of fascinating 2D and 3D supramolecular architectures. From a theoretical point of view, both the σ-hole approach and the NBO analysis applied to the [Au(phen)Br_2_]^+^ and [Br_3_]^−^ building blocks represent tools capable of rationalizing the resulting supramolecular architectures and to account for subtle structural effects. Further studies are ongoing in our laboratory aimed at isolating different related supramolecular systems based on diimine-dihalido complexes.

## Data Availability

Crystallographic data were deposited at CCCD (CIF deposition number 2205837).

## Supplementary Materials

The following are available online at https://www.mdpi.com/article/10.3390/molecules27196289/s1. Scheme S1: reaction scheme; Figure S1: structural correlation; Figures S2 and S3: intermolecular interactions; Figures S4 and S5: acceptor NBO and MEPS for [Au(phen)Br_2_]^+^ and [Br_3_]^−^; Figure S6: [Au(phen)Br_2_](Br_3_) model adduct with selected bond lengths and angles optimized at DFT level; Tables S1–S3: crystallographic and structural data; Tables S4–S7: DFT results for [Au(phen)Br_2_]^+^.

## References

[1] Williams G.T., Haynes C.J.E., Fares M., Caltagirone C., Hiscock J.R., Gale P.A. (2021). Advances in applied supramolecular technologies. Chem. Soc. Rev..

[2] Gale P. (2000). Supramolecular chemistry: From complexes to complexity. Philos. Trans. R. Soc. A.

[3] Krishnaswamy S., Prusty S., Chartrand D., Hanan G.S., Chand D.K. (2018). Self-Assembled Molecular Squares as Supramolecular Tectons. Cryst. Growth Des..

[4] Sonnenberg K., Mann L., Redeker F.A., Schmidt B., Riedel S. (2020). Polyhalogen and Polyinterhalogen Anions from Fluorine to Iodine. Angew. Chem. Int. Ed..

[5] Tebbe K.F., Buchem R. (1997). The Most Iodine-Rich Polyiodide Yet: Fe_3_I_29_. Angew. Chem. Int. Ed..

[6] Svensson H., Kloo L.L. (2003). Synthesis, Structure, and Bonding in Polyiodide and Metal Iodide−Iodine Systems. Chem. Rev..

[7] Aragoni M.C., Arca M., Demartin F., Devillanova F.A., Garau A., Isaia F., Lippolis V., Rizzato S., Verani G. (2004). [Ni(L)(MeCN)]^2+^ complex cation as a template for the assembly of extended I_3_^−^·I_5_^−^ and I_5_^−^·I_7_^−^ polyiodide networks {L=2,5,8-trithia [9](2,9)-1,10-phenanthrolinophane}. Synthesis and structures of [Ni(L)(MeCN)]I_8_ and [Ni(L)(MeCN)]I_12_. Inorg. Chim. Acta.

[8] Haller H., Riedel S. (2014). Recent Discoveries of Polyhalogen Anions–from Bromine to Fluorine. Z. Anorg. Allg. Chem..

[9] Aragoni M.C., Arca M., Devillanova F.A., Hursthouse M.B., Huth S.L., Isaia F., Lippolis V., Mancini A., Ogilvie H. (2005). Self-assembly of supramolecular architectures based on polybromide anions: Crystal structure of [H_4_tppz^4+^](Br^−^)_2_(Br_4_^2−^) [tppz = tetra(2-pyridyl)pyrazine]. Inorg. Chim. Acta.

[10] Aragoni M.C., Arca M., Devillanova F.A., Isaia F., Lippolis V. (2012). Adducts of S/Se Donors with Dihalogens as a Source of Information for Categorizing the Halogen Bonding. Cryst. Growth Des..

[11] Easton M.E., Ward A.J., Hudson T., Turner P., Masters A.F., Maschmeyer T. (2015). The Formation of High-Order Polybromides in a Room-Temperature Ionic Liquid: From Monoanions ([Br_5_]^−^ to [Br_11_]^−^) to the Isolation of [PC_16_H_36_]_2_[Br_24_] as Determined by van der Waals Bonding Radii. Chem. Eur. J..

[12] Korobeynikov N.A., Usoltsev A.N., Novikov A.S., Abramov P.A., Sokolov M.N., Adonin S.A. (2022). Selenium(IV) Polybromide Complexes: Structural Diversity Driven by Halogen and Chalcogen Bonding. Molecules.

[13] Bondi A. (1964). van der Waals Volumes and Radii. J. Phys. Chem..

[14] Sonnenberg K., Pröhm P., Müller C., Beckers H., Steinhauer S., Lentz D., Riedel S. (2018). Closing the Gap: Structural Evidence for the Missing Hexabromide Dianion [Br_6_]^2−^. Chem. Eur. J..

[15] Wolff M., Meyer J., Feldmann C. (2011). [C_4_MPyr]_2_[Br_20_]: Ionic-Liquid-Based Synthesis of a Three-Dimensional Polybromide Network. Angew. Chem..

[16] Aragoni M.C., Arca M., Devillanova F.A., Isaia F., Lippolis V., Mancini A., Pala L., Slawin A.M.Z., Woollins J.D. (2003). First example of an infinite polybromide 2D-network. Chem. Commun..

[17] Cavallo G., Metrangolo P., Milani R., Pilati T., Priimagi A., Resnati G., Terraneo G. (2016). The Halogen Bond. Chem. Rev..

[18] Lim J.Y.C., Beer P.D. (2018). Sigma-Hole Interactions in Anion Recognition. Chem.

[19] Palusiak M. (2010). On the nature of halogen bond—The Kohn–Sham molecular orbital approach. J. Mol. Struct. THEOCHEM.

[20] Kellett C.W., Kennepohl P., Berlinguette C.P. (2020). π Covalency in the halogen bond. Nat. Commun..

[21] Metrangolo P., Meyer F., Pilati T., Resnati G., Terraneo G. (2008). Halogen Bonding in Supramolecular Chemistry. Angew. Chem..

[22] Arca M., Ciancaleoni G., Pintus A., Lippolis V., Santi C., Lenardão E.J., Braga A.L. (2022). Computational Methods to Study Chalcogen Bond. Chalcogen Chemistry: Fundamentals and Applications.

[23] Oliveira V., Kraka E., Cremer D. (2016). The intrinsic strength of the halogen bond: Electrostatic and covalent contributions described by coupled cluster theory. Phys. Chem. Chem. Phys..

[24] Anderson L.N., Aquino F.W., Raeber A.E., Chen X., Wong B.M. (2018). Halogen Bonding Interactions: Revised Benchmarks and a New Assessment of Exchange vs Dispersion. J. Chem. Theory Comput..

[25] Dong W., Li Q., Scheiner S. (2018). Comparative Strengths of Tetrel, Pnicogen, Chalcogen, and Halogen Bonds and Contributing Factors. Molecules.

[26] Bauzá A., Frontera A. (2020). Halogen and Chalcogen Bond Energies Evaluated Using Electron Density Properties. ChemPhysChem.

[27] Aragoni M.C., Arca M., Devillanova F.A., Hursthouse M.B., Huth S.L., Isaia F., Lippolis V., Mancini A., Ogilvie H.R., Verani G. (2005). Reactions of pyridyl donors with halogens and interhalogens: An X-ray diffraction and FT-Raman investigation. J. Organomet. Chem..

[28] Aragoni M.C., Arca M., Devillanova F.A., Hursthouse M.B., Huth S.L., Isaia F., Lippolis V., Mancini A. (2004). Square-pyramidal bonding of I_2_ molecules at the I^−^ nodes of a polyiodide infinite pseudo-cubic 3D-network. Cryst. Eng. Comm..

[29] Blake A.J., Gould R.O., Parsons S., Radek C., Schröder M. (1995). Self-Assembly of Polyanions at a Metal Cation Template: Syntheses and Structures of [{Ag([18]aneS_6_)}I_7_]_n_ and [Ag([18]aneS_6_)]I_3_. Angew. Chem. Int. Ed. Engl..

[30] Martínez-Camarena Á., Savastano M., Blasco S., Delgado-Pinar E., Giorgi C., Bianchi A., García-España E., Bazzicalupi C. (2022). Assembly of Polyiodide Networks with Cu(II) Complexes of Pyridinol-Based Tetraaza Macrocycles. Inorg. Chem..

[31] Schneider D., Schuster O., Schmidbaur H. (2005). Bromination of (phosphine)gold(I) bromide complexes: Stoichiometry and structure of products. Dalton Trans..

[32] Döring C., Jones P.G. (2016). Two-Dimensional Networks of [AuCl_4_]^−^ and [AuBr_4_]^−^Anions. Z. Anorg. Allg. Chem..

[33] Lu W., Tung Chan K., Wu S.-X., Chen Y., Che C.-M. (2012). Quest for an intermolecular Au(III)/Au(III) interaction between cyclometalated gold(III) cations. Chem. Sci..

[34] Koskinen L., Jääskeläinen S., Kalenius E., Hirva P., Haukka M. (2014). Role of C–H···Au and Aurophilic Supramolecular Interactions in Gold–Thione Complexes. Cryst. Growth Des..

[35] Chernyshev A.N., Chernysheva M.V., Hirva P., Kukushkin V.Y., Haukka M. (2015). Weak aurophilic interactions in a series of Au(III) double salts. Dalton Trans..

[36] Taouss C., Jones P.G. (2011). Halogenation of (phosphine chalcogenide)gold(I) halides; some unexpected products. Dalton Trans..

[37] Rimmer E.L., Bailey R.D., Pennington W.T., Hanks T.W. (1998). The reaction of iodine with 9-methylacridine: Formation of polyiodide salts and a charge-transfer complex. J. Chem. Soc. Perkin Trans. 2.

[38] Sharafie D., Amani V., Naseh M. (2018). Synthesis, spectroscopic characterization, crystal structure determination and DFT calculations of [Au(Me_2_phen)Br_2_][AuBr_2_]. Chem. Pap..

[B39-molecules-27-06289] 39.CSD codes: BOFSEV, EREGIT, EREGOZ, HAZMOL, JEBQUF, NUTCUD, QIRRAK, SOKCED, WOFKIM, XIZDIW, XIZDOC, XIZDUI, XIZFAQ, XIZFIY, and ZEQPES.

[B40-molecules-27-06289] 40.A search on the CCSD reveals 253 hits of isolated tribromide anions with Br–Br mean distances of 2.54(6) Å and Br–Br–Br mean angle of 178(2)°.

[41] Powell B.M., Heal K.M., Torrie B.H. (1984). The temperature dependence of the crystal structures of the solid halogens, bromine and chlorine. Mol. Phys..

[42] Gilli G., Gilli P. (2009). The Nature of the Hydrogen Bond: Outline of a Comprehensive Hydrogen Bond Theory.

[43] Reed A.E., Weinstock R.B., Weinhold F. (1985). Natural-population analysis. J. Chem. Phys..

[44] Reed A.E., Weinhold F. (1985). Natural Localized Molecular Orbitals. J. Chem. Phys..

[45] Desiraju G.R., Parthasarathy R. (1989). The Nature of Halogen-Halogen Interactions: Are Short Halogen Contacts Due to Specific Attractive Forces or Due to Close Packing of Nonspherical Atoms?. J. Am. Chem. Soc..

[46] Dumitrescu D., Shova S., Man I.C., Caira M.R., Popa M.M., Dumitrascu F. (2020). 5-Iodo-1-Arylpyrazoles as Potential Benchmarks for Investigating the Tuning of the Halogen Bonding. Crystals.

[47] Salmasi R., Gholizadeh M., Salimi A., Garrison J.C. (2016). The synthesis of 1,2-ethanediylbis(triphenylphosphonium) ditribromide as a new brominating agent in the presence of solvents and under solvent-free conditions. J. Iran. Chem. Soc..

[48] Stang S., Lebkucher A., Walter P., Kaifer E., Himmel H.-J. (2012). Redox-Active Guanidine Ligands with Pyridine and p-Benzoquinone Backbones. Eur. J. Inorg. Chem..

[49] Bondarenko M.A., Novikov A.S., Fedin V.P., Sokolov M.N., Adonin S.A. (2020). The stabilization of decabromide {Br_10_}^2−^ anion in the structure of Sb(V) bromide complex. J. Coord. Chem.

[50] Adonin S.A., Bondarenko M.A., Novikov A.S., Abramov P.A., Plyusnin P.E., Sokolov M.N., Fedin V.P. (2019). Halogen bonding-assisted assembly of bromoantimonate(V) and polybromide-bromoantimonate-based frameworks. CrystEngComm.

[51] Reed A.E., Curtiss L.A., Weinhold F. (1988). Intermolecular interactions from a natural bond orbital, donor-acceptor viewpoint. Chem. Rev..

[52] Robertson K.N., Bakshi P.K., Cameron T.S., Knop O. (1997). Polyhalide Anions in Crystals. The Br_8_^2−^ anion in diquinuclidinium octabromide, the crystal structures of Me_4_PBr_3_ and quinuclidinium tribromide, and Ab initio calculations on polybromide anions. Z. Anorg. Allg. Chem..

[53] Sheldrick G. (2014). SHELXT: Integrating space group determination and structure solution. Acta Cryst..

[54] Sheldrick G.M. (2008). A short history of SHELX. Acta Cryst..

[55] Frisch M.J., Trucks G.W., Schlegel H.B., Scuseria G.E., Robb M.A., Cheeseman J.R., Scalmani G., Barone V., Petersson G.A., Nakatsuji H. (2016). Gaussian 16.

[56] Pintus A., Aragoni M.C., Bellec N., Devillanova F.A., Lorcy D., Isaia F., Lippolis V., Randall R.A.M., Slawin A.M.Z., Woollins J.D. (2012). Structure-Property Relationships in Pt^II^ Diimine-Dithiolate Nonlinear Optical Chromophores Based on Arylethylene-1,2-Dithiolate and 2-Thioxothiazoline-4,5-Dithiolate. Eur. J. Inorg. Chem..

[57] Pintus A., Aragoni M.C., Isaia F., Lippolis V., Lorcy D., Slawin A.M.Z., Woollins J.D., Arca M. (2015). On the Role of Chalcogen Donor Atoms in Diimine-Dichalcogenolate Pt^II^ SONLO Chromophores: Is It Worth Replacing Sulfur with Selenium?. Eur. J. Inorg. Chem..

[58] Aragoni M.C., Arca M., Binda M., Caltagirone C., Lippolis V., Natali D., Podda E., Sampietro M., Pintus A. (2021). Platinum Diimine-Dithiolate Complexes as a New Class of Photoconducting Compounds for Pristine Photodetectors: Case Study on [Pt(Bipy)(Naph-Edt)] (Bipy = 2,2′-Bipyridine; Naph-Edt^2−^ = 2-Naphthylethylene-1,2-Dithiolate). Dalton Trans..

[59] Adamo C., Barone V. (1999). Toward Reliable Density Functional Methods without Adjustable Parameters: The PBE0 Model. J. Chem. Phys..

[60] Weigend F., Ahlrichs R. (2005). Balanced Basis Sets of Split Valence, Triple Zeta Valence and Quadruple Zeta Valence Quality for H to Rn: Design and Assessment of Accuracy. Phys. Chem. Chem. Phys..

[61] Ross R.B., Powers J.M., Atashroo T., Ermler W.C., LaJohn L.A., Christiansen P.A. (1998). Ab Initio Relativistic Effective Potentials with Spin–Orbit Operators. IV. Cs through Rn. J. Chem. Phys..

[62] Pritchard B.P., Altarawy D., Didier B., Gibson T.D., Windus T.L. (2019). New Basis Set Exchange: An Open, Up-to-Date Resource for the Molecular Sciences Community. J. Chem. Inf. Model..

[63] Dennington R.D., Keith T.A., Millam J.M. (2016). GaussView, 6.0. 16.

[64] Skripnikov L.V. (2017). Chemissian, Version 4.53, Visualization Computer Program. https://www.chemissian.com.

