# Supplementary material for: Self-Assembly of Supramolecular Architectures Driven by σ-Hole Interactions: A Halogen-Bonded 2D Network Based on a Diiminedibromido Gold(III) Complex and Tribromide Building Blocks
Source: Molecules. 2022 Sep 23;27(19):6289. doi: 10.3390/molecules27196289 (PMC9571214; doi:10.3390/molecules27196289)
Supplement: Supplementary file 1 [file molecules-27-06289-s001.zip › molecules-1931951-supplementary new.pdf]

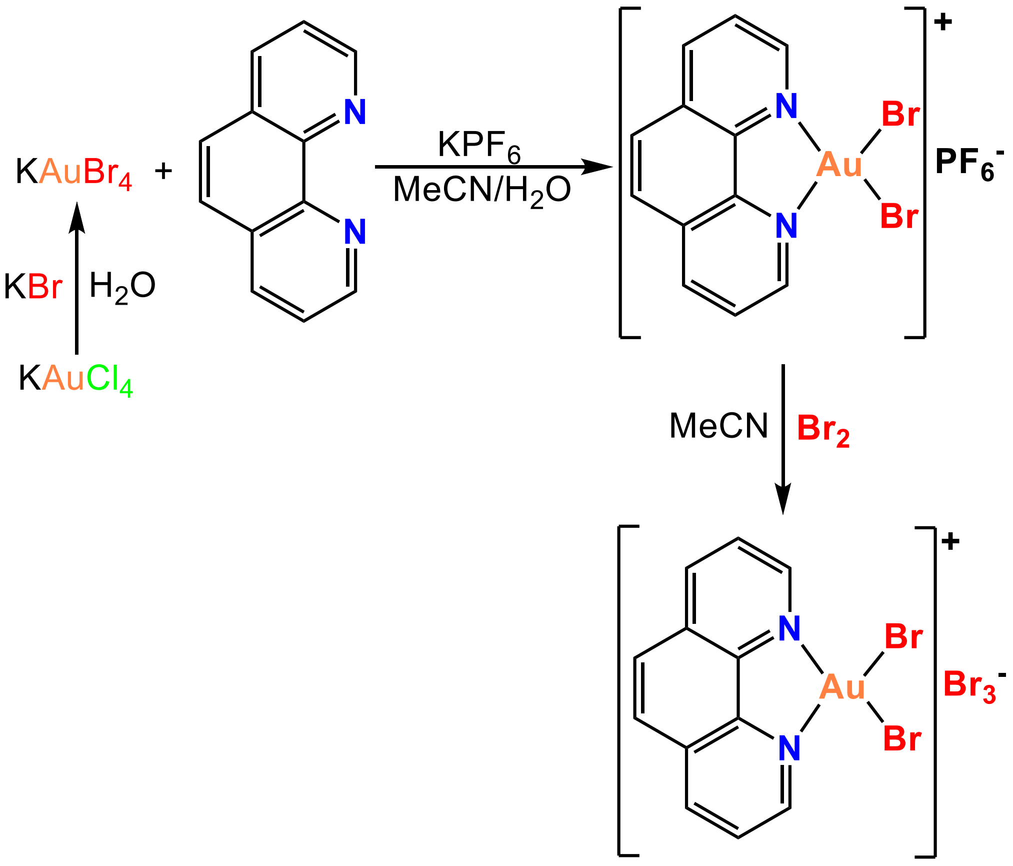

1

Scheme S1. Reaction scheme for the synthesis of $\{[Au(phen)Br_2](Br_3)\}_\infty$ (compound **1**).

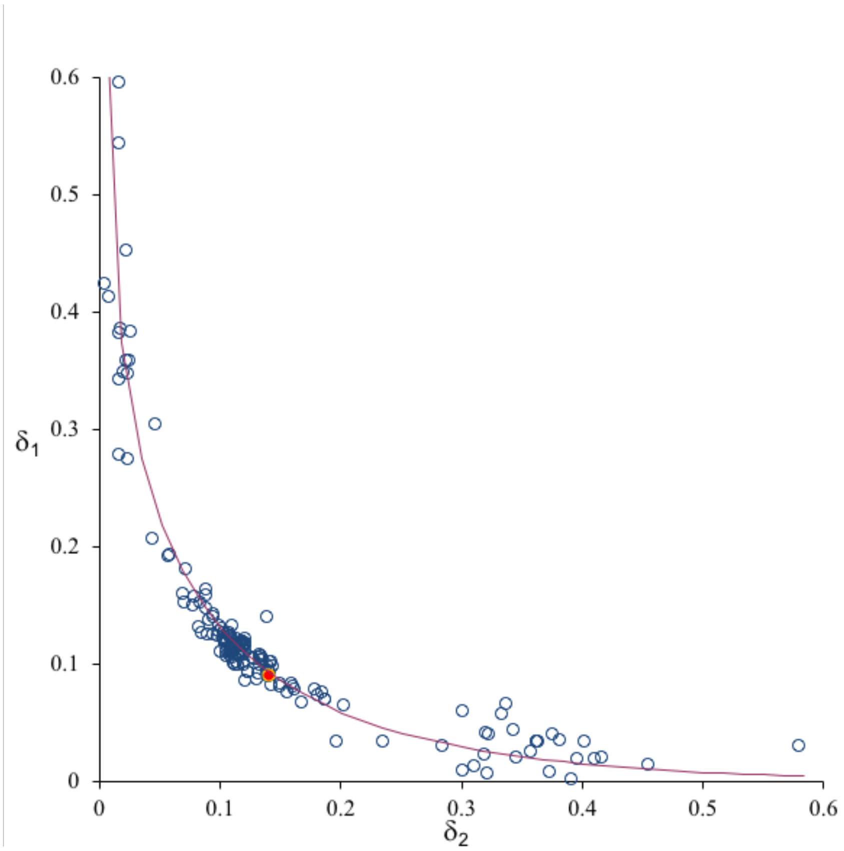

Figure S1. Structural data of linear Br1–Br2–Br3 fragments deposited at the CSD reported as scatter plot of δ_1 *vs.* δ_2, where δ_1 = δ(Br1–Br2) and δ_2 = δ(Br2–Br3). Structural data were taken from M. C. Aragoni et al., *Cryst. Growth Des*. 2012, **12**, 2769−2779. The red dot represents the data for the tribromide anion reported in this work. The solid curve represents the least-squares fit of all data according to the following equation:

$$\delta_1 = -k \cdot ln\left[1 - e^{-\frac{\delta_2}{k}}\right]$$

Fitted parameter: k = 0.164, RMSD = 0.029, normalized RMSD = 0.048; for all data: k = 0.158; RMSD = 0.046; normalized RMSD = 0.061.

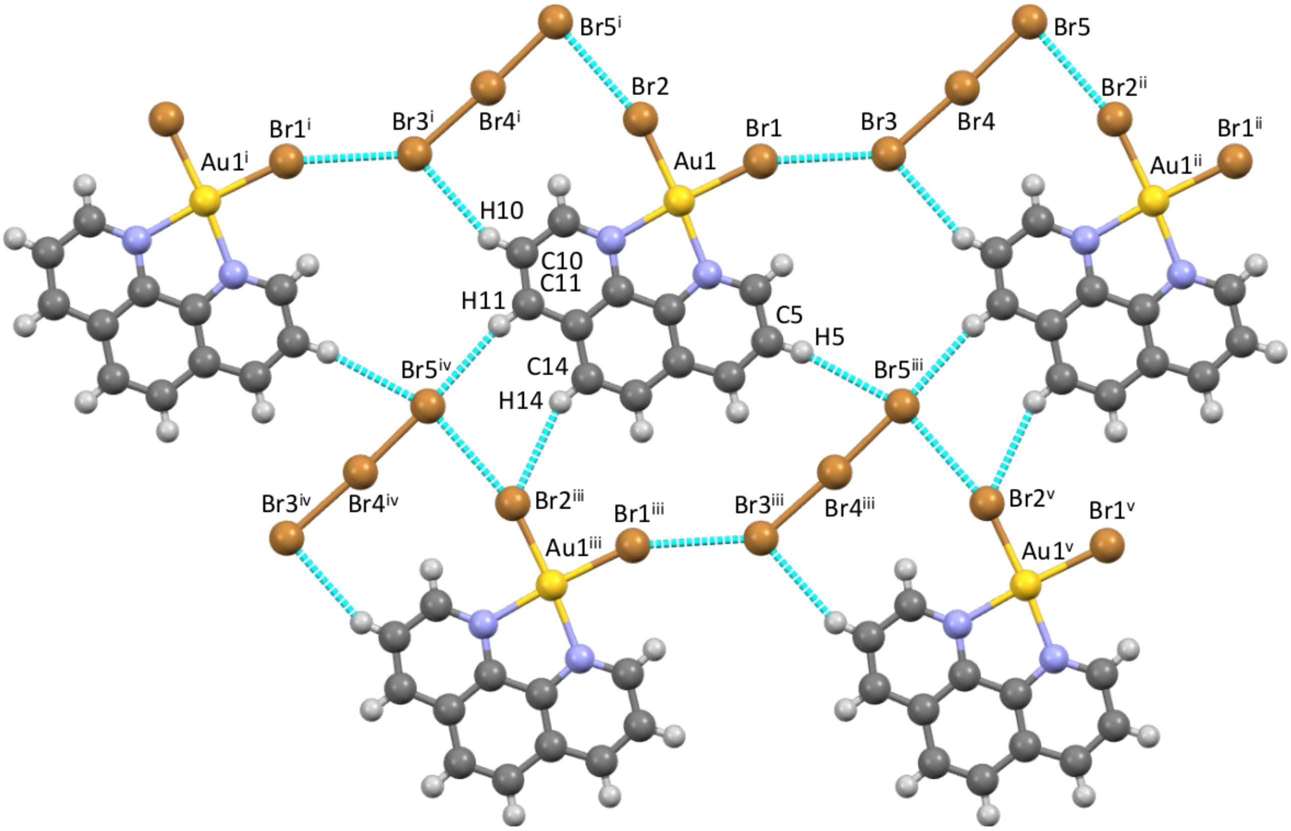

Figure S2. C–H···Br and Br···Br interactions forming a 2D-network laying on plane *ac* in compound **1**: C10–H10···Br3^{i}, 2.972 (C10···Br3^{i}, 3.852); C11–H11···Br5^{iv}, 2.830 (C11···Br5^{iv} 3.765); C14–H14···Br2^{iii}, 2.942 (C14···Br2^{iii}, 3.830); C5–H5···Br5^{iii}, 2.997(C5 ···Br5^{iii}, 3.933) Å. Symmetry operations: ^{i} = x, y, 1-z; ^{ii} = x, y, 1+z; ^{iii} = 1+x, y, z; ^{iv} = 1+x, y, -1+z; ^{v} = 1+x, y, 1+z.

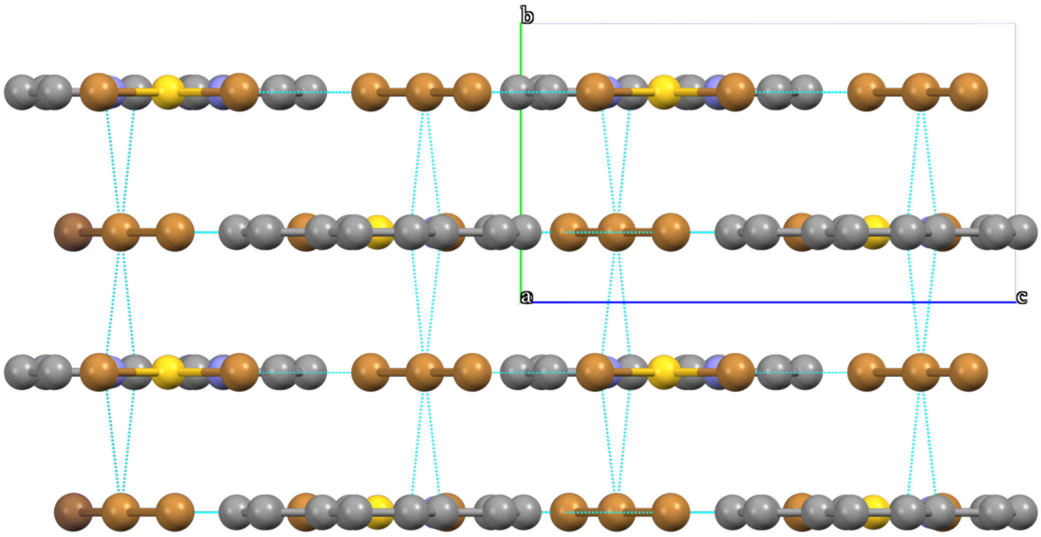

Figure S3. Portion of the crystal packing of compound **1** seen along the *a* axis: C7···Br4^{vi}, 3.430; C12···Br4^{vi}, 3.418 Å; ^{vi} = 1-x, -1/2+y, 1-z; sum of C and Br vdW radii 3.60 Å.

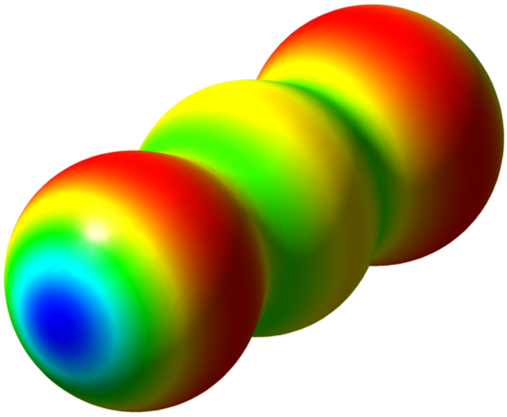

Figure S4. Potential electrostatic energy map [range -0.172 (red) – 0.120 (blue) a.u.] calculated for the tribromide anion at DFT level on the density map (electron density = $9.0 \cdot 10^{-3}$ |e|/Bohr^3).

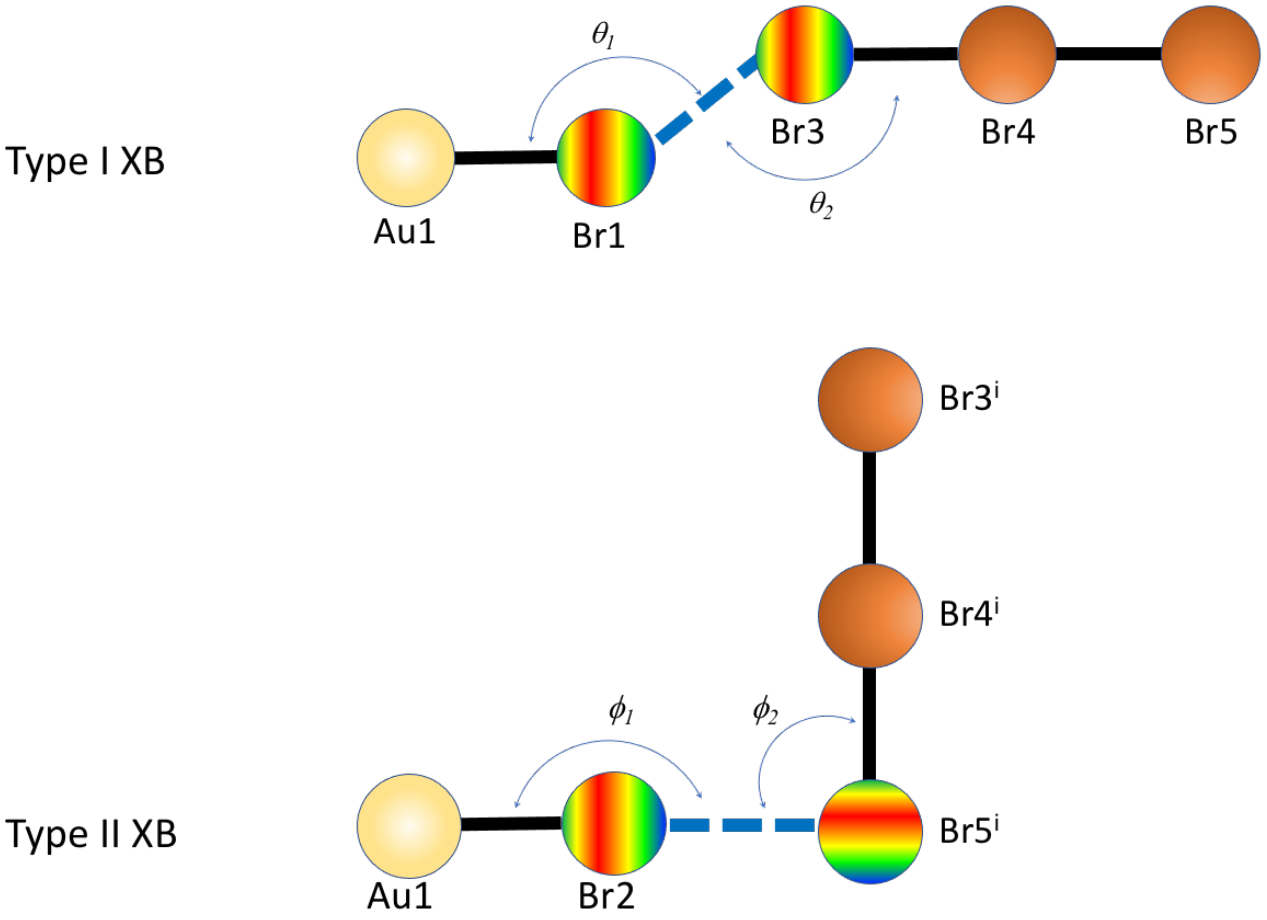

Figure S5. Schematic representation of type-I (top) and type-II (bottom) XB interactions in compound **1** in terms of electrostatic potential (red = negative potential; blue = positive potential). θ_1 = 157.4°, θ_2 = 141.5°, ϕ_1 = 164.2°, ϕ_2 = 86.7°.

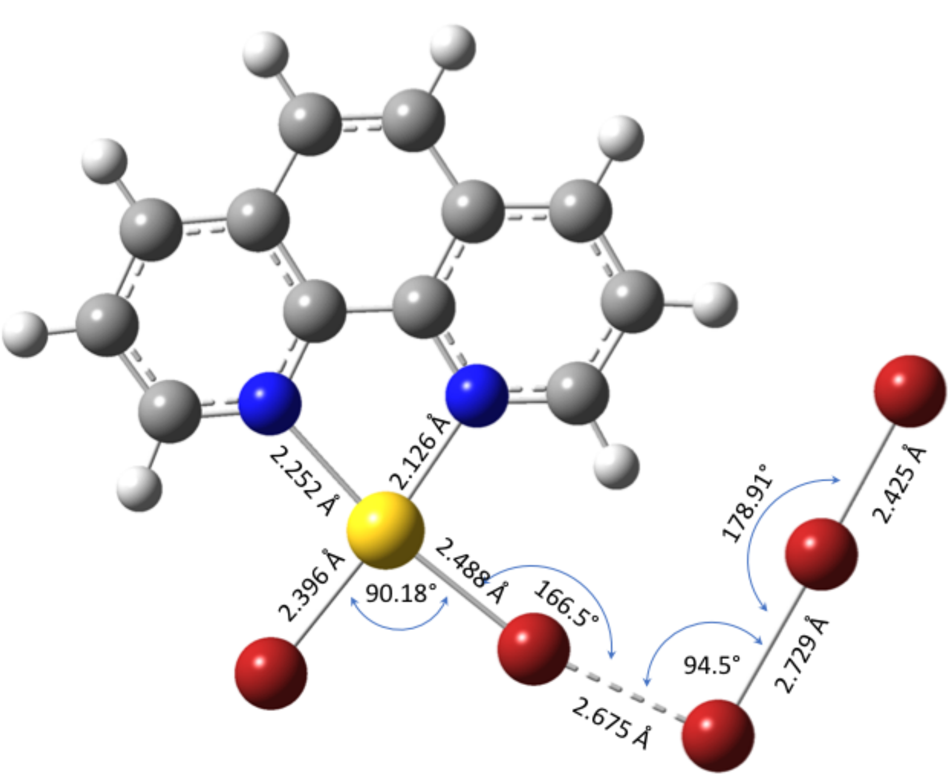

Figure S6. $[Au(phen)Br_2](Br_3)$ model adduct with selected bond lengths and angles optimized at DFT level.

Table S1. Crystal data and refinement parameters for compound **1**.

Formula	$C_{12}H_8AuBr_5N_2$
$D_{calc.}$/ g cm^{-3}	3.046
m/mm^{-1}	20.542
Formula Weight	776.70
Colour	yellow
Shape	platelet-shaped
Size/mm^3	0.05×0.02×0.01
T/K	93
Crystal System	monoclinic
Space Group	$P2_1/m$
a/Å	10.657(2)
b/Å	6.7010(12)
c/Å	12.495(3)
α/°	90
β/°	108.404(4)
γ/°	90
V/Å^3	846.7(3)
Z	2
Z'	0.5
Wavelength/Å	0.71075
Radiation type	Mo K_α
Θ_{min}/°	1.718
Θ_{max}/°	25.357
Measured Reflections.	25164
Independent Reflections	1682
Reflections I≥2 σ(I)	1492
R_{int}	0.0941
Parameters	121
Restraints	0
Largest Peak	0.91
Deepest Hole	-0.89
GooF	0.927
wR_2 (all data)	0.0408
wR_2	0.0406
R_1 (all data)	0.0215
R_1	0.0197

Table S2. Bond Lengths in Å for compound **1**.

Au1−N8	2.077(4)
Au1−N1	2.074(4)
Au1−Br2	2.3825(7)
Au1−Br1	2.3853(8)
Br3−Br4	2.6022(9)
Br4−Br5	2.4871(9)
N1−C6	1.326(7)
N1−C2	1.369(7)
N8−C9	1.338(7)
N8−C7	1.352(7)
C2−C3	1.395(8)
C2−C7	1.436(8)
C3−C4	1.392(9)
C3−C13	1.434(10)
C4−C5	1.367(10)
C5−C6	1.397(9)
C7−C12	1.407(8)
C9−C10	1.373(9)
C10−C11	1.364(9)
C11−C12	1.398(9)
C12−C14	1.430(9)
C13−C14	1.350(10)

Table S3. Bond Angles in ° for compound **1**.

N8−Au1−N1	81.11(18)
N8−Au1−Br2	94.49(13)
N1−Au1−Br2	175.59(13)
N8−Au1−Br1	176.06(13)
N1−Au1−Br1	94.96(13)
Br2−Au1−Br1	89.45(3)
Br5−Au1−Br3	177.32(3)
C6−N1−C2	119.7(5)
C6−N1−Au1	128.7(4)
C2−N1−Au1	111.6(4)
C9−N8−C7	119.8(5)
C9−N8−Au1	128.2(4)
C7−N8−Au1	112.0(4)
N1−C2−C3	122.4(5)
N1−C2−C7	117.5(5)
C3−C2−C7	120.0(5)
C2−C3−C4	116.6(6)
C2−C3−C13	118.1(6)
C4−C3−C13	125.3(6)
C5−C4−C3	120.8(6)
C4−C5−C6	119.7(6)
N1−C6−C5	120.8(6)
N8−C7−C12	121.7(5)
N8−C7−C2	117.8(5)
C12−C7−C2	120.5(5)
N8−C9−C10	121.0(6)
C9−C10−C11	120.5(6)
C10−C11−C12	119.9(6)
C11−C12−C7	117.0(6)
C11−C12−C14	124.6(6)
C7−C12−C14	118.4(6)
C14−C13−C3	122.4(6)
C13−C14−C12	120.7(6)

Table S4. Orthogonal Cartesian coordinates for the cation $[Au(phen)Br_2]^+$ at the DFT-optimized geometry.

Center Number	Atomic Number	Coordinates (Å) X	Y	Z
1	79	4.222095	5.025750	3.422568
2	35	2.557442	5.025750	5.133540
3	35	2.473822	5.025750	1.797143
4	7	5.853772	5.025750	4.724570
5	7	5.786264	5.025750	2.040289
6	6	7.035443	5.025750	4.061582
7	6	8.264026	5.025750	4.735484
8	6	8.225044	5.025750	6.141241
9	1	9.152320	5.025750	6.703301
10	6	7.015043	5.025750	6.787921
11	1	6.952382	5.025750	7.868270
12	6	5.832169	5.025750	6.043881
13	1	4.853394	5.025750	6.510656
14	6	6.999651	5.025750	2.642988
15	6	5.698184	5.025750	0.723660
16	1	4.697115	5.025750	0.306862
17	6	6.842027	5.025750	-0.078786
18	1	6.725229	5.025750	-1.154630
19	6	8.083140	5.025750	0.506240
20	1	8.980847	5.025750	-0.101942
21	6	8.192900	5.025750	1.908165
22	6	9.467667	5.025750	3.968641
23	1	10.415315	5.025750	4.494353
24	6	9.433570	5.025750	2.613612
25	1	10.353626	5.025750	2.040942

Table S5. Summary of Natural Population Analysis for the cation $[Au(phen)Br_2]^+$ at the DFT-optimized geometry.

			Natural Population			
Atom	No	Natural Charge (|e|)	Core	Valence	Rydberg	Total
Au	1	0.51790	67.99445	10.45333	0.03432	78.48210
Br	2	-0.11758	27.99894	7.10245	0.01619	35.11758
Br	3	-0.11754	27.99894	7.10241	0.01619	35.11754
N	4	-0.38075	1.99920	5.34083	0.04072	7.38075
N	5	-0.38075	1.99920	5.34083	0.04072	7.38075
C	6	0.15886	1.99899	3.82040	0.02176	5.84114
C	7	-0.06644	1.99906	4.04960	0.01778	6.06644
C	8	-0.08907	1.99914	4.07213	0.01780	6.08907
H	9	0.24829	0.00000	0.75047	0.00124	0.75171
C	10	-0.22250	1.99919	4.20558	0.01774	6.22250
H	11	0.25923	0.00000	0.73976	0.00100	0.74077
C	12	0.10099	1.99926	3.87820	0.02155	5.89901
H	13	0.25812	0.00000	0.74061	0.00127	0.74188
C	14	0.15893	1.99899	3.82033	0.02176	5.84107
C	15	0.10090	1.99926	3.87828	0.02155	5.89910
H	16	0.25814	0.00000	0.74059	0.00127	0.74186
C	17	-0.22251	1.99919	4.20559	0.01774	6.22251
H	18	0.25923	0.00000	0.73977	0.00100	0.74077
C	19	-0.08903	1.99914	4.07209	0.01780	6.08903
H	20	0.24829	0.00000	0.75047	0.00124	0.75171
C	21	-0.06646	1.99906	4.04962	0.01778	6.06646
C	22	-0.15472	1.99915	4.13851	0.01706	6.15472
H	23	0.24657	0.00000	0.75229	0.00114	0.75343
C	24	-0.15469	1.99915	4.13848	0.01706	6.15469
H	25	0.24658	0.00000	0.75229	0.00114	0.75342

Table S6. Orthogonal Cartesian coordinates for neutral adduct $[Au(phen)Br_2](Br_3)$ at the DFT-optimized geometry.

Center Number	Atomic Number	Coordinates (Angstroms) X	Y	Z
1	6	-0.004145	-0.005449	0.010035
2	6	0.008028	0.018620	1.437305
3	6	1.238297	0.021602	2.113151
4	6	2.454865	0.000495	1.359221
5	6	2.411598	-0.023032	-0.045478
6	6	1.146668	-0.025464	-0.703372
7	6	3.642977	-0.043021	-0.722512
8	6	4.815217	-0.038996	-0.009474
9	6	4.760897	-0.014850	1.388732
10	7	3.616863	0.003994	2.034455
11	6	-1.163183	0.039794	2.214719
12	6	-1.070261	0.062250	3.580744
13	6	0.195110	0.063664	4.176761
14	7	1.306229	0.044005	3.466025
15	79	3.278201	0.043730	4.260076
16	35	2.529843	0.088800	6.632508
17	35	5.565521	0.039905	4.973827
18	35	1.153680	0.137057	8.925340
19	35	-1.290257	0.137716	7.710831
20	35	-3.440985	0.137519	6.590390
21	1	3.653897	-0.061508	-1.806905
22	1	5.776824	-0.054074	-0.506085
23	1	5.655565	-0.010498	2.004999
24	1	0.303899	0.081243	5.256844
25	1	-1.943723	0.079154	4.223531
26	1	-2.130277	0.038127	1.723679
27	1	1.124174	-0.043661	-1.787056
28	1	-0.964078	-0.007357	-0.493730

Table S7. Summary of Natural Population Analysis for the cation [Au(phen)Br_2](Br_3) at the DFT-optimized geometry.

			Natural Population			
Atom	No	Natural Charge	Core	Valence	Rydberg	Total
C	1	-0.16407	1.99915	4.14755	0.01737	6.16407
C	2	-0.08016	1.99906	4.06239	0.01870	6.08016
C	3	0.16183	1.99899	3.81719	0.02198	5.83817
C	4	0.16174	1.99900	3.81713	0.02212	5.83826
C	5	-0.07911	1.99906	4.06131	0.01874	6.07911
C	6	-0.17330	1.99914	4.15676	0.01740	6.17330
C	7	-0.12046	1.99914	4.10343	0.01789	6.12046
C	8	-0.23588	1.99918	4.21849	0.01820	6.23588
C	9	0.08485	1.99927	3.89344	0.02244	5.91515
N	10	-0.40070	1.99929	5.36067	0.04074	7.40070
C	11	-0.11351	1.99913	4.09628	0.01809	6.11351
C	12	-0.22673	1.99917	4.20877	0.01879	6.22673
C	13	0.09540	1.99926	3.88329	0.02205	5.90460
N	14	-0.40597	1.99923	5.36689	0.03986	7.40597
Au	15	0.44632	67.99516	10.53869	0.01983	78.55368
Br	16	-0.08770	27.99932	7.06747	0.02091	35.08770
Br	17	-0.28958	27.99905	7.27537	0.01516	35.28958
Br	18	-0.21420	27.99991	7.19988	0.01442	35.21420
Br	19	-0.04561	27.99991	7.01346	0.03223	35.04561
Br	20	-0.25577	27.99987	7.23870	0.01720	35.25577
H	21	0.23117	0.00000	0.76750	0.00134	0.76883
H	22	0.24049	0.00000	0.75843	0.00108	0.75951
H	23	0.24146	0.00000	0.75722	0.00132	0.75854
H	24	0.26290	0.00000	0.73404	0.00305	0.73710
H	25	0.27172	0.00000	0.72641	0.00187	0.72828
H	26	0.23291	0.00000	0.76574	0.00135	0.76709
H	27	0.23036	0.00000	0.76839	0.00125	0.76964
H	28	0.23160	0.00000	0.76717	0.00123	0.76840
